# Survey dataset on workplace incivility, emotional exhaustion and adaptive performance among employees working in the front line: A case study

**DOI:** 10.1016/j.dib.2023.109497

**Published:** 2023-08-13

**Authors:** Nurdiyana Sumri, Daniella M. Mokhtar

**Affiliations:** Centre for Research in Psychology and Human Well-being, Faculty of Social Sciences and Humanities, Universiti Kebangsaan Malaysia, Malaysia

**Keywords:** Workplace incivility, Emotional exhaustion, Adaptive performance, Frontline workers

## Abstract

The data belongs to a sample of 201 frontline workers in Malaysia. This demographic data was collected using a cross-sectional questionnaire via an online survey and analyzed using SPSS version 25. This data was used to investigate the relationship between workplace incivility, emotional exhaustion and adaptive performance (handling emergencies, handling work stress, creative problem solving, learning new tasks, technology and procedure and demonstrating interpersonal adaptability) among frontline workers. The analyzed data will be useful in contributing to further research into the effects of workplace incivility on employees’ well-being and job performance. It will also give insights to stakeholders and those at managerial level who formulate appropriate intervention plans to overcome or reduce the issue of workplace bullying among frontline workers.

Specifications Table

**Table:** 

Subject	Applied Psychology, Psychology
Specific subject area	Industrial and Organizational Psychology, Occupational Psychology, Counterproductive Work Behavior (CWB)
Type of data	Table
How the data were acquired	Data was acquired using a cross-sectional survey of 201 respondents. The survey consisted of demographic characteristics, workplace incivility, emotional exhaustion, and adaptive performance.
Data format	Raw and descriptive
Description of data collection	The data was collected using the snowball sampling technique via the researcher's personal social media platforms such as Facebook, WhatsApp, Telegram, Instagram, LinkedIn, and email. Respondents were required to answer the survey via a Google Form.
Data source location	· Institution: Universiti Kebangsaan Malaysia · City/Town/Region: UKM Bangi · Country: Malaysia · Latitude and longitude 2.917325, 101.783719
Data accessibility	Repository name: Mendeley Data Data identification number: 10.17632/6v9g8dn4jz.1 Direct URL to data: https://data.mendeley.com/datasets/6v9g8dn4jz

## 1 Value of the Data


 

• The dataset explores job-related issues in the workplace (i.e., workplace incivility) and the relationship with employee performance and emotional exhaustion.
• This dataset adds to the scarce literature on workplace incivility in Malaysia and opens the door to further study on the subject.
• This data can be used by other researchers for sampling in different job scopes.
• Managers at various levels can utilize the dataset as a guide for developing employee development programs that address workplace incivility in their organizations.

## 2 Objective

Workplace incivility is common in some public and private sector organisations in Malaysia [7]. Employee well-being, the psychological contract, job satisfaction, and work engagement were all negatively impacted by workplace incivility [6]. The dataset is intended to help researchers better understand workplace incivility and its relationship with front-line employees’ emotional exhaustion and job performance in Malaysian contexts.

## 3 Data Description

The data provided includes the response rate (Table 1), demographic information (Table 2), and descriptive values of the variables used to measure workplace incivility, emotional exhaustion, and adaptive performance (Table 3). A total of 205 questionnaires were collected via an online survey of frontline workers in the health, education, finance, information technology, agriculture, manufacturing, construction, and business sectors in Malaysia. Due to redundant data, four questionnaires were removed. Therefore, 201 of the 205 questionnaires were accepted (98.05%). Table 2 highlights the demographic characteristics of the respondents. Table 3 displays the descriptive statistics relating to the variables and their sub-dimensions. The skewness of workplace incivility was found to be 1.42, indicating that the distribution was right-skewed. The kurtosis of workplace incivility was found to be 1.59, indicating that the distribution was more heavy-tailed compared to the normal distribution. As for emotional exhaustion, the skewness was found to be 0.41, indicating that the distribution was right-skewed. The kurtosis of emotional exhaustion was found to be −0.51, indicating that the distribution was more light-tailed compared to the normal distribution. Meanwhile, for adaptive performance, the skewness was −0.12, indicating that the distribution was left-skewed. The kurtosis of adaptive performance was found to be −0.52, indicating that the distribution was more light-tailed compared to the normal distribution. Together with the analyzed data, two versions of the raw data have been shared as research data in Data Mendeley (https://data.mendeley.com/datasets/6v9g8dn4jz): SPSS (WorkplaceIncivility.sav) and Excel (WorkplaceIncivility.xlsx). The data contains variables from the questionnaire, for example responses of the Workplace Incivility Scale Item (WIS1-WIS7), Emotional Exhaustion Scale (EE1-EE9) and Adaptive Performance (API1-API20). Demographic information is included in the raw data (gender, age, race, marital status, educational background, rank, year of service, employment sector and location). Moreover, the survey questions in Malay and English have been included as supplementary material (DIB English.docx and DIB Malay.docx).

**Table 1:** Rate of response to the administered questionnaire. Source: Researcher's Online Survey, 2021.

Questionnaire		Number of respondents	Rate of response (%)
Administered	205		
Not Completed		0	0
Completed		205	100.00
Usable		201	98.05
Not usable		4	1.95
Total	205	205	100.00

**Table 2:** Demographic characteristics of respondents. Source: Researcher's Online Survey, 2021.

Demographic Characteristics	Items	Frequency	Percentage (%)
Gender	Male	59	29.4
	Female	142	70.6
Age	20–29	47	23.4
	30–39	113	56.2
	40–49	26	12.9
	50–59	15	7.5
Race	Malay	183	91.0
	Chinese	8	4.0
	Indian	4	2.0
	Others	6	3.0
Education Background	SPM	19	9.5
	Pre-University/Matriculation/Certificate	6	3.0
	STPM	3	1.5
	Diploma	38	18.9
	Degree	111	55.2
	Master/PhD	24	11.9
Marital Status	Single	59	29.4
	Married	136	67.7
	Divorced	6	3.0
Rank	Support	96	47.8
	Professional and Management	105	52.2
Years of Service	1–5 years	59	29.4
	6–10 years	55	27.4
	11–15	50	24.9
	16–20	9	4.5
	>20		
Employment sector	Health	86	42.8
	Education	72	35.8
	Services	21	10.4
	Security	13	6.5
	Agriculture	2	1.0
	Information Technology	2	1.0
	Manufacturing and Construction	3	1.5
	Business	2	1.0
Location	Urban	158	78.6
	Rural	43	21.4

**Table 3:** Descriptive statistics of the variables contained in the dataset Source: Researcher's Online Survey, 2021.

Variable	N	Min	Max	Mean		Std. Deviation	Skewness		Kurtosis	
				Statistic	Std. Error		Statistic	Std. Error	Statistic	Std. Error
Workplace incivility	201	7	43	14.32	.57	8.01	1.42	.17	1.59	.34
Emotional Exhaustion	201	9	45	21.96	.56	7.93	.41	.17	−0.51	.34
Adaptive Performance	201	60	105	85.00	.76	10.82	−0.12	.17	−0.52	.34
Handling emergencies	201	8	20	16.44	.19	2.63	−0.54	.17	−0.06	.34
Handling work stress	201	9	20	15.51	.18	2.61	−0.12	.17	−0.52	.34
Creative problem solving	201	12	25	20.07	.22	3.06	−0.12	.17	−0.52	.34
Learning new tasks	201	7	15	12.48	.14	1.93	−0.17	.17	−0.67	.34
Demonstrating interpersonal adaptability	201	10	20	16.56	.16	2.25	−0.34	.17	−0.10	.34

## 4 Experimental Design, Materials and Methods

The dataset contains information on the relationship between workplace incivility, emotional exhaustion, and adaptive performance among frontline workers. A cross-sectional online survey method was used for data collection. The researcher used the convenient and snowball sampling techniques because they enabled the selection of respondents who would be appropriate for and specific to this study. These methods are particularly effective when studying certain populations, including those who encounter sensitive issues like workplace incivility [8]. The sample included workers from several sectors in Malaysia, such as health, education, security, services, manufacturing, information technology, finance, and business. The questionnaire was created using items adapted from previous research [1], [2], [3], [4], [5], [6], [7], [8], [9], [10], [11], [12] and it was back-translated into Malay. The survey was divided into four sections: demographic questions (Section A), workplace incivility (Section B), emotional exhaustion (Section C) and adaptive performance (Section D). For section A, information was collected on the demographic backgrounds of the respondents, such as their age, gender, race, marital status, academic background, work sector, years of service, rank and work location. The collected data was analyzed using IBM SPSS Version 25.0. Descriptive, frequency and reliability tests were used.

Section B includes seven items adapted from the Workplace Incivility Scale (WIS-7) developed by Cortina et al [9]. The Workplace Incivility Scale (WIS) was used in the current study to measure impolite behavior - such as disrespect, impoliteness, or condescension behavior performed by superiors or colleagues in the workplace, as experienced by the respondents. The WIS uses a 7-point Likert scale to measure workplace incivility with a continuum of 1 to 7, on which 1 means “never”, 2 means “very rarely”, 3 means “rarely”, 4 means “once in a while”, 5 means “sometimes”, 6 means “frequently” and 7 means “very frequently” as well as a score range of 7 (1 × 7) to 49 (7 × 7). All the items are positive. Among the WIS items are as follows; ― Paid little attention to your statement or showed little interest in your opinion?, ― Ignored or excluded you from professional camaraderie?, ― Doubted your judgment on a matter over which you have responsibility?

Section C consists of nine items adapted from the Emotional Exhaustion Scale (EES) instrument by Maslach & Jackson [3]. It was used to measure the level of emotional exhaustion of the respondents. This instrument is one of the three subscales of the Maslach Burnout Inventory (emotional exhaustion, depersonalization, and reduction of personal achievement), which originally contained 22 original items. The emotional exhaustion scale contains 9 items characterize feelings of emotional exhaustion resulting from work. This scale's scoring method is a 5-point Likert Scale with a continuum of 1 to 5, where 1 is Strongly Disagree and 2 is Disagree. 3: Uncertain, 4: Agree, and 5: Strongly Agree as well as a score range of 9 (1 × 9) to 45 (5 × 9). All items are positive. Examples of EES items include; ―I feel used up at the end of the workday, ―Working with people all day is really a strain for me, ―I feel like I am at the end of my rope.

Section D consists of 20 items adapted from the Adaptive Performance Inventory self-assessment. This was translated by Fatimah wati Halim et al [10] from the Adaptive Performance Inventory by developed Pulakos et al [4] and Charbonnier-Voirin & Roussel [1]. This instrument consists of five dimensions of adaptive performance, namely handling emergencies, handling work stress, creative problem solving, learning new tasks, and demonstrating interpersonal adaptation. This instrument uses a 5-point Likert scale with an overall score range of 20 (1 × 20), up to a maximum score of 100 (5 × 20). All the items are positive. Examples of the items include― I quickly decide on the actions to take to resolve problems (handling emergencies) ― I keep my cool in situations where I am required to make many decisions (handling stress), ― I develop new tools and methods to resolve new problems (creative problem solving) ― I am on the lookout for the latest innovations in my job to improve the way I work (learning new tasks)― I learn new ways to do my job better in order to collaborate with such people (interpersonal adaptability).

The respondents were briefed on the background and purpose of the research before answering the questionnaire. They were also informed that their participation was voluntary, and anonymous, as well as being informed that they could opt out of the survey at any time. To complete the survey, they had to answer all the questions. Data was collected using Google Forms and extracted into Excel format. It was then run through the Statistical Package for Social Sciences (SPSS) and analyzed using descriptive and inferential statistics (Table 1, Table 2, Table 3, Table 4).

**Table 4:** Cronbach's alpha value for each construct.

Construct	No. of questions	Cronbach's alpha
Workplace Incivility Scale	7	.938
Emotional Exhaustion Scale	9	.891
Adaptive Performance Inventory	20	.938

Table 4 shows the Cronbach's alpha value of each instruments. For WIS, the alpha value is 0.938; for EES, the alpha value is 0.891, and for API, the alpha value is 0.938.

## Ethics statements

Data was anonymised, and no personal information, such as phone numbers or email addresses, was requested. Informed consent was obtained from all the individual participants included in the data collection process. Before answering the questionnaire, the participants were briefed on the objectives of the data collection and their responses were anonymous and confidential. They were given the option to withdraw at any point during the study. The IRB number for this project is UKM PPI/111/8/JEP-2021-548.

## CRediT authorship contribution statement

**Nurdiyana Sumri:** Writing – original draft, Writing – review & editing. **Daniella M. Mokhtar:** Supervision, Writing – review & editing.

## Declaration of Competing Interest

The authors declare that they have no known competing financial interests or personal relationships that could have appeared to influence the work reported in this paper.

## Data Availability

Workplace incivility, emotional exhaustion and adaptive performance (Original data) (Mendeley Data)

## Appendix Supplementary materials

Image, application 1

Image, application 2
