# Supplementary material for: Survey dataset on workplace incivility, emotional exhaustion and adaptive performance among employees working in the front line: A case study
Source: Data Brief. 2023 Aug 13;50:109497. doi: 10.1016/j.dib.2023.109497 (PMC10470363; doi:10.1016/j.dib.2023.109497)
Supplement: Supplementary file 1 [file mmc1.docx]

**SURVEY ON WORKPLACE INCIVILITY, EMOTIONAL EXHAUSTION AND ADAPTIVE PERFORMANCE**

**SECTION A: DEMOGRAPHIC QUESTIONS**

1. Age : ......................(years)
2. Gender : Male ( )

Female ( )

1. Race : Malay ( )

Chinese ( )

Indian ( )

Other race ( )

Please state:........................

1. Marital status : Single ( )

Married ( )

Divorced ( )

1. Highest education : SPM ( )

STPM ( )

Pre-University/Matriculation/Certificate ( )

Diploma ( )

Degree ( )

Masters/PhD ( )

1. Employment sector : Health ( )

Education ( )

Security ( )

Hospitality ( )

Services ( )

Others (Please state) ................

1. Rank : Support ( )

Professional and managerial ( )

1. Tenure : 1-5 years ( )

6-10 years ( )

11-15 years ( )

16-20 years ( )

> 20 years ( )

1. Location : Urban ( )

Rural ( )

**SECTION B: WORKPLACE INCIVILITY**

During the past six months, have you been in a situation where any of your superiors or coworkers...

| **1** | **2** | **3** | **4** | **5** | **6** | **7** |
| --- | --- | --- | --- | --- | --- | --- |
| **Never** | **Very rarely (Once in several months)** | **Rarely (At least one a month)** | **Once in a while**  **(Several times in a month)** | **Sometimes (At least once a week)** | **Frequently (Several times a week)** | **Very frequently (Several times a day)** |

| No | Item | 1 | 2 | 3 | 4 | 5 | 6 | 7 |
| --- | --- | --- | --- | --- | --- | --- | --- | --- |
| 1. | Put you down or was condescending to you? |  |  |  |  |  |  |  |
| 2. | Paid little attention to your statement or showed little interest in your opinion? |  |  |  |  |  |  |  |
| 3. | Made demeaning or derogatory remarks about you? |  |  |  |  |  |  |  |
| 4. | Addressed you in unprofessional terms, either publicly or privately? |  |  |  |  |  |  |  |
| 5. | Ignored or excluded you from professional camaraderie? |  |  |  |  |  |  |  |
| 6. | Doubted your judgment on a matter over which you have responsibility? |  |  |  |  |  |  |  |
| 7. | Made unwanted attempts to draw you into a discussion of personal matters? |  |  |  |  |  |  |  |

**SECTION C: EMOTIONAL EXHAUSTION**

This section contains 9 questions. Please tick (/) according to choices of answers given below.

| **1** | **2** | **3** | **4** | **5** |
| --- | --- | --- | --- | --- |
| **Strongly Disagree** | **Disagree** | **Uncertain** | **Agree** | **Strongly Agree** |

| No. | Item | 1 | 2 | 3 | 4 | 5 |
| --- | --- | --- | --- | --- | --- | --- |
| 1. | I feel emotionally drained by my work. |  |  |  |  |  |
| 2. | I feel used up by the end of the day. |  |  |  |  |  |
| 3. | I feel fatigued when I have to get up in the morning to face another day on the job. |  |  |  |  |  |
| 4. | Working with people all day is really a strain for me. |  |  |  |  |  |
| 5. | Working with people directly puts too much stress on me. |  |  |  |  |  |
| 6. | I feel ‘burned out’ from my work. |  |  |  |  |  |
| 7. | I feel frustrated by my job. |  |  |  |  |  |
| 8. | I feel I’m working too hard in my job. |  |  |  |  |  |
| 9. | I feel like I’m at the end of my rope. |  |  |  |  |  |

**SECTION D: ADAPTIVE PERFORMANCE**

This section contains 20 questions. Please tick (/) according to choices of answers given below.

| **1** | **2** | **3** | **4** | **5** |
| --- | --- | --- | --- | --- |
| **Strongly Disagree** | **Disagree** | **Uncertain** | **Agree** | **Strongly Agree** |

| No | Item | 1 | 2 | 3 | 4 | 5 |
| --- | --- | --- | --- | --- | --- | --- |
| 1. | I am able to achieve total focus on the situation to act quickly |  |  |  |  |  |
| 2. | I quickly decide on the actions to take to resolve problems |  |  |  |  |  |
| 3. | I analyze possible solutions and their ramifications quickly to select the most appropriate one |  |  |  |  |  |
| 4. | I easily reorganize my work to adapt to the new circumstances |  |  |  |  |  |
| 5. | I feel at easy even though my tasks change and occur at fast pace |  |  |  |  |  |
| 6. | I keep my cool in situations where I am required to make many decisions |  |  |  |  |  |
| 7. | I look for solutions by having a calm discussion with colleagues |  |  |  |  |  |
| 8. | My colleagues ask for my advice regularly when situations are difficult because of my self-control |  |  |  |  |  |
| 9. | I develop new tools and methods to resolve new problems |  |  |  |  |  |
| 10. | I use a variety of sources/types of information to come up with an innovative solution |  |  |  |  |  |
| 11. | I do not hesitate to go against established ideas and propose an innovative solution |  |  |  |  |  |
| 12. | I am on the lookout for the latest innovations in my job to improve the way I work |  |  |  |  |  |
| 13. | I undergo training on a regular basis at or outside of work to keep my competencies up to date |  |  |  |  |  |
| 14. | I prepare for change by participating in every project or assignment that enables me to do so |  |  |  |  |  |
| 15. | I look for every opportunity that enables me to improve my performance (training, group project, exchanges with colleagues, etc.) |  |  |  |  |  |
| 16. | I learn new ways to do my job better in order to collaborate with such people. |  |  |  |  |  |
| 17. | Within my department, people rely on me to suggest new solution |  |  |  |  |  |
| 18. | I willingly adapt my behaviour whenever I need to in order to work well with others |  |  |  |  |  |
| 19. | Developing good relationships with all my counterparts is an important factor of my effectiveness |  |  |  |  |  |
| 20. | I try to understand the viewpoints of my counterparts to improve my interaction with them |  |  |  |  |  |
