# Supplementary material for: Survey dataset on workplace incivility, emotional exhaustion and adaptive performance among employees working in the front line: A case study
Source: Data Brief. 2023 Aug 13;50:109497. doi: 10.1016/j.dib.2023.109497 (PMC10470363; doi:10.1016/j.dib.2023.109497)
Supplement: Supplementary file 2 [file mmc2.docx]

**Soal Selidik Ketidaksopanan Tempat Kerja, Keletihan Emosi dan Prestasi Adaptif**

**BAHAGIAN A: MAKLUMAT DEMOGRAFI**

1. Umur : ......................(tahun)
2. Jantina : Lelaki ( )

Perempuan ( )

1. Bangsa : Melayu ( )

Cina ( )

India ( )

Lain-lain ( )

1. Status perkahwinan : Bujang ( )

Berkahwin ( )

Duda/Janda ( )

1. Pendidikan tertinggi : SPM ( )

STPM ( )

Pra-Universiti/Matrikulasi/Sijil ( )

Diploma ( )

Sarjana Muda ( )

Sarjana/Doktof Falsafah ( )

1. Sektor pekerjaan : Kesihatan ( )

Pendidikan ( )

Keselamatan ( )

Hospitaliti ( )

Perkhidmatan ( )

Lain-lain (nyatakan) ................

1. Jawatan : Pelaksana ( )

Pengurusan dan Profesional ( )

1. Tempoh bekerja : 1-5 tahun ( )

6-10 tahun ( )

11-15 tahun ( )

16-20 tahun ( )

> 20 tahun ( )

1. Lokasi : Bandar ( )

Luar Bandar ( )

**BAHAGIAN B: KETIDAKSOPANAN TEMPAT KERJA**

Dalam tempoh 6 bulan lepas, anda pernah berada dalam keadaan di mana penyelia atau rakan sekerja anda ...

| **1** | **2** | **3** | **4** | **5** | **6** | **7** |
| --- | --- | --- | --- | --- | --- | --- |
| **Tidak Pernah** | **Jarang-jarang Sekali (Sekali dalam beberapa bulan)** | **Jarang-jarang (Sekurang-kurangnya sekali sebulan)** | **Sekali-sekala (Sekurang-kurangnya beberapa kali sebulan)** | **Kadang-kadang (Sekurang-kurangnya sekali seminggu)** | **Kerap (Beberapa kali dalam seminggu)** | **Sangat Kerap (Beberapa kali dalam sehari)** |

| **No** | **Item** | **1** | **2** | **3** | **4** | **5** | **6** | **7** |
| --- | --- | --- | --- | --- | --- | --- | --- | --- |
| 1. | Membuat anda kecewa atau telah merendah-rendahkan anda? |  |  |  |  |  |  |  |
| 2. | Kurang menumpukan perhatian kepada kenyataan anda atau menunjukkan minat yang sedikit terhadap pendapat anda? |  |  |  |  |  |  |  |
| 3. | Membuat kenyataan atau membuat komen-komen menghina tentang anda? |  |  |  |  |  |  |  |
| 4. | Memanggil anda dalam istilah yang tidak profesional, sama ada secara umum atau persendirian? |  |  |  |  |  |  |  |
| 5. | Mengabaikan atau mengecualikan anda daripada persahabatan profesional? |  |  |  |  |  |  |  |
| 6. | Meragui keputusan anda mengenai perkara yang anda dipertanggungjawabkan? |  |  |  |  |  |  |  |
| 7. | Membuat percubaan yang tidak diingini untuk menarik anda ke dalam perbincangan perkara-perkara peribadi. |  |  |  |  |  |  |  |

**BAHAGIAN C: KELETIHAN EMOSI**

Bahagian ini mengandungi 9 soalan. Sila tandakan (/) berdasarkan pilihan jawapan di bawah.

| **1** | **2** | **3** | **4** | **5** |
| --- | --- | --- | --- | --- |
| **Sangat Tidak Setuju** | **Agak Tidak Setuju** | **Tidak Setuju Mahu Pun Setuju** | **Agak Setuju** | **Sangat Setuju** |

| **No.** | **Item** | **1** | **2** | **3** | **4** | **5** |
| --- | --- | --- | --- | --- | --- | --- |
| 1. | Saya rasa lemah emosi di tempat kerja. |  |  |  |  |  |
| 2. | Saya rasa kehabisan tenaga selepas waktu kerja. |  |  |  |  |  |
| 3. | Saya rasa letih bila bangun pagi dan terpaksa menghadapi satu lagi hari bekerja. |  |  |  |  |  |
| 4. | Bekerja dengan orang lain mendatangkan ketegangan buat saya. |  |  |  |  |  |
| 5. | Bekerja secara langsung dengan orang lain memberi banyak tekanan pada diri saya. |  |  |  |  |  |
| 6. | Saya rasa kehabisan tenaga akibat kerja saya. |  |  |  |  |  |
| 7. | Saya rasa kecewa dengan kerja saya. |  |  |  |  |  |
| 8. | Saya rasa seperti terlalu kuat bekerja. |  |  |  |  |  |
| 9. | Saya rasa seperti berada di penghujung kerjaya. |  |  |  |  |  |

**BAHAGIAN D: PRESTASI ADAPTIF**

Bahagian ini mengandungi 20 soalan. Sila tandakan (/) berdasarkan pilihan jawapan di bawah.

| **1** | **2** | **3** | **4** | **5** |
| --- | --- | --- | --- | --- |
| **Sangat Tidak Setuju** | **Agak Tidak Setuju** | **Tidak Setuju Mahu Pun Setuju** | **Agak Setuju** | **Sangat Setuju** |

| **No** | **Item** | **1** | **2** | **3** | **4** | **5** |
| --- | --- | --- | --- | --- | --- | --- |
| 1. | Saya terus fokus kepada keadaan semasa (sewaktu peristiwa itu berlaku) untuk bertindak balas dengan cepat. |  |  |  |  |  |
| 2. | Saya segera mengambil tindakan yang berkesan untuk menyelesaikan masalah. |  |  |  |  |  |
| 3. | Saya memeriksa pilihan yang ada dan implikasinya untuk memilih penyelesaian yang terbaik. |  |  |  |  |  |
| 4. | Saya mudah menukar rancangan untuk berhadapan dengan keadaan yang baru. |  |  |  |  |  |
| 5. | Saya tenang apabila berhadapan dengan keadaan sukar atau beban kerja yang sangat mendesak / jadual yang padat |  |  |  |  |  |
| 6. | Saya tidak akan bertindak melulu kepada berita atau situasi yang tidak disangka. |  |  |  |  |  |
| 7. | Saya dapat menguruskan kekecewaan dengan baik menggunakan penyelesaian yang membina tanpa menyalahkan orang lain. |  |  |  |  |  |
| 8. | Rakan-rakan sering mendapatkan nasihat saya apabila mereka berada dalam keadaan yang sukar kerana saya bersikap tenang. |  |  |  |  |  |
| 9. | Saya cuba untuk membentuk kaedah baru untuk menyelesaikan masalah atipikal. |  |  |  |  |  |
| 10. | Saya bergantung kepada pelbagai maklumat untuk mencari penyelesaian inovatif terhadap sebarang masalah. |  |  |  |  |  |
| 11. | Saya berusaha untuk menangani masalah yang berlaku dengan cara mencari penyelesaian yang inovatif. |  |  |  |  |  |
| 12. | Saya mencari inovasi dalam tugas saya supaya dapat memperbaiki kaedah kerja. |  |  |  |  |  |
| 13. | Saya sentiasa mencari peluang untuk memastikan saya mempunyai kemahiran baru. |  |  |  |  |  |
| 14. | Saya menjangka perubahan dalam pekerjaan saya dengan mengambil bahagian dalam tugasan-tugasan yang membantu saya berurusan dengan perubahan semasa. |  |  |  |  |  |
| 15. | Saya sentiasa mencari peluang-peluang (contohnya, latihan, interaksi dengan rakan-rakan, dan lain-lain) yang dapat membantu meningkatkan kualiti diri saya. |  |  |  |  |  |
| 16. | Saya belajar cara baru yang lebih baik di dalam melaksanakan kerja/tugas daripada rakan-rakan. |  |  |  |  |  |
| 17. | Rakan-rakan saya mengambil nasihat saya untuk menjana idea-idea baru dan penyelesaian. |  |  |  |  |  |
| 18. | Saya mengubah cara saya bekerja dengan maklum balas dan cadangan orang lain. |  |  |  |  |  |
| 29. | Saya sentiasa membina hubungan positif dengan orang yang saya berinteraksi apabila menjalankan tugas kerana ia dapat membantu saya melakukan tugas dengan lebih baik. |  |  |  |  |  |
| 20. | Saya cuba untuk mempertimbangkan pandangan orang lain untuk berinteraksi lebih dengan mereka. |  |  |  |  |  |
